# Does contracting of health care in Afghanistan work? Public and service-users' perceptions and experience

**DOI:** 10.1186/1472-6963-11-S2-S11

**Published:** 2011-12-21

**Authors:** Anne Cockcroft, Amir Khan, Noor Md Ansari, Khalid Omer, Candyce Hamel, Neil Andersson

**Affiliations:** 1 CIET Trust Botswana, PO Box 1240, Gaborone, Botswana; 2 CIET in Pakistan, PO Box 13018, Karachi 75350, Pakistan; 3 Department of geography and urban regional planning, University of Peshawar, Peshawar, Pakistan; 4 CIETcanada, 1 Stewart Street, # 319, Ottawa, K1N 6N5, Canada; 5 Centro de Investigación de Enfermedades Tropicales, Universidad Autónoma de Guerrero, Acapulco, Mexico

## Abstract

**Background:**

In rebuilding devastated health services, the government of Afghanistan has provided access to basic services mainly by contracting with non-government organisations (NGOs), and more recently the Strengthening Mechanism (SM) of contracting with Provincial Health Offices. Community-based information about the public's views and experience of health services is scarce.

**Methods:**

Field teams visited households in a stratified random sample of 30 communities in two districts in Kabul province, with health services mainly provided either by an NGO or through the SM and administered a questionnaire about household views, use, and experience of health services, including payments for services and corruption. They later discussed the findings with separate community focus groups of men and women. We calculated weighted frequencies of views and experience of services and multivariate analysis examined the related factors.

**Results:**

The survey covered 3283 households including 2845 recent health service users. Some 42% of households in the SM district and 57% in the NGO district rated available health services as good. Some 63% of households in the SM district (adjacent to Kabul) and 93% in the NGO district ordinarily used government health facilities. Service users rated private facilities more positively than government facilities. Government service users were more satisfied in urban facilities, if the household head was not educated, if they had enough food in the last week, and if they waited less than 30 minutes. Many households were unwilling to comment on corruption in health services; 15% in the SM district and 26% in the NGO district reported having been asked for an unofficial payment. Despite a policy of free services, one in seven users paid for treatment in government facilities, and three in four paid for medicine outside the facilities. Focus groups confirmed people knew payments were unofficial; they were afraid to talk about corruption.

**Conclusions:**

Households used government health services but preferred private services. The experience of service users was similar in the SM and NGO districts. People made unofficial payments in government facilities, whether SM or NGO run. Tackling corruption in health services is an important part of anti-corruption measures in Afghanistan.

## Background

Public health services in Afghanistan were in near total collapse in 2001. Services have since improved, but much remains to be done [1,2]. A household survey in 2006 indicated improvements in under-five and infant mortality rates compared with figures from 2000 [3]. A key focus has been on improving access of the population to basic health services. The government developed a Basic Package of Health Services (BPHS) in 2002, revised in 2005 [4], and arranged access to these basic services mainly through contracting non-governmental organisations (NGOs) to provide the services, in collaboration with donor partners [2]. Subsequently, under a scheme known as the Ministry of Public Health Strengthening Mechanism (MOPH-SM), the central MOPH contracted Provincial Health Offices (PHOs) to provide the BPHS in some provinces, in the same way as NGOs were awarded contracts [2]. In 2007, an estimated 82% of the population lived in districts covered by contracts to provide the BPHS; this does not necessarily mean that 82% of the population actually had access to or used health facilities providing the BPHS [2]. According to official policy in 2008, services in government health facilities (including those run directly by the MOPH, and those provided under contract by the SM or by a contracted NGO) were provided free of charge.

Given the difficult security situation in many parts of the country, reliable representative community-based data about use and experience of health services in Afghanistan are hard to collect. Most of the information about the functioning of the health services is facilities-based (collected from the facility staff and records and from the users of the facility). Repeated cycles of a survey of facilities across the country have shown improvements over time in many functions and in staff views and experience, as well as high and improving ratings of the service from patients using the facilities [5]. However, a 2006 review of progress in health services pointed out that having facilities on the ground and able to provide the BPHS does not mean that everyone has access to or chooses to use these services, and concluded that “Research at the community and household level is very important to determine other reasons why some people do, and others do not, make use of the health services available” [^2^]. The people who do not make use of available public health services are often the most disadvantaged.

In order to provide information about the use and experience of health services from the viewpoint of households, in 2008 the government of Afghanistan, through the MOPH, commissioned a demonstration social audit of health services in two districts, both in Kabul province. In the first district (the NGO district), in the north of the province, the district hospital and most of the five basic health centres (BHCs) were run by a contracted NGO. In the second district (the SM district), adjacent to Kabul city, the two comprehensive health centres (CHCs) and most of the BHCs were run under the MOPH-SM scheme. This paper describes the social audit process, its findings, and their implications for service provision. We discussed the findings with stakeholders in Afghanistan soon after the data collection and analysis but they have not been previously published.

## Methods

The Institutional Review Board of the MOPH reviewed the project proposal and gave ethical approval in May 2008 (ref 244874).

### The sample

In consultation with the MOPH, we purposively selected two districts for the demonstration social audit, one with government health services run by a contracted NGO (the NGO district) and one with these services run by the Provincial Health Office contracted to do so under the Strengthening Mechanism (the SM district). Both districts are in Kabul province, and the SM district is adjacent to Kabul. The NGO district covers some 1200 sq Km and the SM district some 2500 sq Km. A group drawn from the project steering committee and district representatives estimated 35% of the population in the SM district and 10% in the NGO district lived in urban communities, with a small nomad population in each district. The group updated the list of communities in the two districts and categorised each one as urban (including semi-urban), rural or nomad. We drew a stratified random sample of 15 communities from each district: one nomad community for each district; five urban communities in the SM district and two in the NGO district; and nine rural communities in the SM district and 12 in the NGO district.

In each sample community, the field team surveyed around 120 contiguous households having at least one child under the age of five years. Radiating from a randomly selected starting point, they visited households until they reached the target number. There was no sub-sampling within the sites.

To ensure the sample proportions of urban and rural (including nomad) reflected those estimated in the population, we calculated site weights and applied these when calculating district estimates.

### Instruments and data collection

We developed the instruments for the survey based on priority issues identified by the MOPH. We translated them into Dari and Pashto, with back translation to check for any loss of meaning during translation. The *household questionnaire* included questions about demographics and socio-economic status of the household. It enquired about perceptions and use of health services (government and other) and asked the most recent service users (or someone answering on their behalf, especially for children) about their experience of the service most recently used, including waiting times, availability of medicines, and payments. We did not ask whether service users had previously used any other health service for the same problem. We piloted the questionnaire in non-sample urban and rural settings in and around Kabul, and made minor adjustments to improve interpretation and flow. The final version included up to 94 items (in most cases less than this) and took about 30 minutes to administer.

A three day training for two field teams in each district included classroom sessions and field practices with the instruments. Data collection took place from July to August 2008. Security guards accompanied each field team to the survey sites. They were familiar with the local areas, and they guided the field teams to reach the communities and assisted them to move around in the communities.

In November 2008, after preliminary analysis of findings from the household survey, we developed a guide to feedback and discuss findings from the household survey with separate focus groups of men and women. Trained teams returned to all the 30 communities of the household survey and facilitated separate male and female focus group discussions in each community: a total of 60 groups, each with 8-10 participants, mainly from among the households covered in the household survey. In each group a trained reporter made notes of the discussion in Dari, later expanded as necessary in discussion with the group facilitator.

### Analysis

Data entry relied on Epi-Info, the public domain data entry and analysis package. Double data entry and verification of discordant records minimised key-stroke errors. Further cleaning looked for logical inconsistencies and out of range responses. Analysis relied on CIETmap open source software that combines epidemiological analysis with raster and vector mapping [6]. Initial analysis generated weighted frequencies of the main indicators about perceptions, use, and experience of health services. The analysis of experience and perceptions of recent service users focused on those who had visited a health facility within the last six months. Further analysis examined factors related to outcomes of interest, in bivariate analysis and then in multivariate analysis using the Mantel Haenszel procedure [7] with an adjustment for clustering described by Gilles Lamothe, based on a variance estimator to weight the Mantel Haenszel odds ratio for cluster-correlated data [8,9]. Multivariate analysis began with saturated models including all variables potentially associated with the outcome, and stepped down to final models including only variables that remained significantly associated with the outcome. Initial models for outcomes for households included: age, sex, and education of the respondent; education of the household head; poverty status of the household (whether they had enough food in the last week); urban or rural location; and district. Initial models for outcomes for service users included: age and sex of the service user; poverty status of their household; government or private facility; urban or rural location; and district. When there was important heterogeneity between districts we undertook separate models for each district. Associations are expressed as the adjusted Odds Ratio (ORa) and its cluster-adjusted 95% confidence interval (CIca).

A small group including Dari speakers reviewed the focus group reports, identified recurring themes in the discussions, and extracted relevant quotes.

## Results

The trained field teams collected data from 1639 households in the SM district and 1644 households in the NGO district. Table 1 shows household characteristics in the two districts. In the NGO district, there were more rural households, household heads were less educated and households were poorer.

**Table 1:** Characteristics of the sample households in the two districts

Characteristic	Fraction (weighted %) of households	
	SM District	NGO District
*Location*		
Urban/semi-urban	518/1639 (32)	204/1644 (11)
Rural/nomad	1121/1639 (68)	1521/1644 (89)
Male household head	1600/1639 (98)	1449/1644 (88)
Household head primary education or above	687/1588 (43)	265/1365 (19)
Household income sufficient for expenditures	964/1633 (59)	779/1641 (48)
Enough food in house in last week	1067/1621 (66)	810/1622 (50)

In SM district government health services were provided mainly by contracting the Provincial Health Office under the Strengthening Mechanism; in NGO district the services were provided mainly by a contracted NGO.

### Household perceptions of quality and corruption in health services

**Figure 1:**
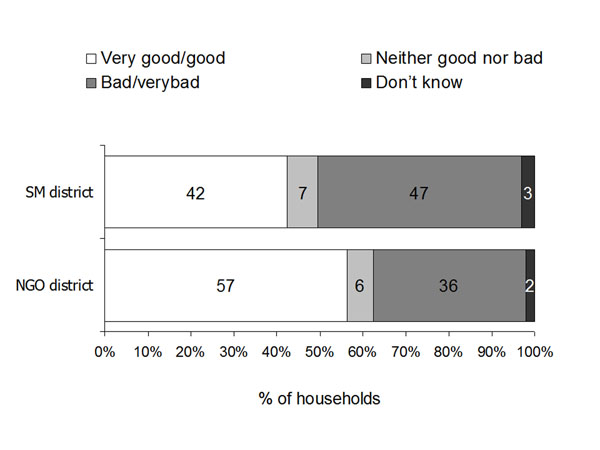
**Household ratings of available health services in the two districts**. In SM district government health services were provided mainly by contracting the Provincial Health Office under the Strengthening Mechanism; in NGO district the services were provided mainly by a contracted NGO.

Figure 1 shows the household perceptions about available health services in the two districts. Household ratings in the NGO district tended to be more positive than in the SM district but the difference between the two districts in the proportion of households rating health services as good or very good was not significant at the 5% level. In the SM district, in the final multivariate model, households reporting enough food in the last week (i.e. not the poorest households) were more likely to rate available health services as good, compared with households without enough food in the last week. This effect was stronger in rural areas (ORa 3.95, 95% CI_ca_ 1.94-8.06) than in urban areas (ORa 2.10, 95% CI_ca_ 1.08-4.08). In the NGO district, none of the household level variables was significantly associated with the household rating of available health services, nor was there a significant difference between urban and rural sites.

Focus groups of men and women voiced a number of complaints about health services available to them, including lack of access to facilities, lack of medicines and equipment, and poor treatment in facilities.

“What can I say, sister? Everybody is treated badly at government hospitals.” (Female group, the SM district)

“I went to the hospital but the doctor told me he did not have the equipment to check my blood pressure.” (Female group, the NGO district)

**Figure 2:**
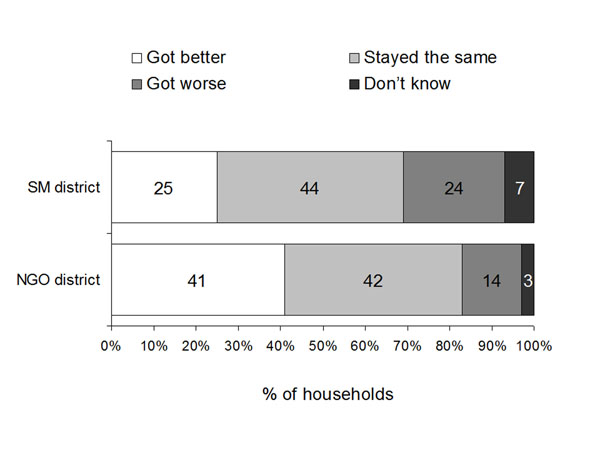
**Household views of changes in health services over last 12 months in the two districts**. In SM district government health services were provided mainly by contracting the Provincial Health Office under the Strengthening Mechanism; in NGO district the services were provided mainly by a contracted NGO.

Figure 2 shows household perceptions of changes in health services in the last 12 months. Respondents in the SM district were significantly less likely than respondents in the NGO district to think health services had improved in the last 12 months (ORa 0.48, 95% CI_ca_ 0.31-0.73). In the SM district, in multivariate analysis, in rural sites only, households with enough food in the last week were more likely to think health services had improved (ORa 3.49, 95% CI_ca_ 2.50-4.88) as were households where the head had some education (ORa 1.69, 95% CI_ca_ 1.21-2.37). In the NGO district, only one factor remained in the final model; households in urban areas were more likely to think services had improved than those in rural areas (ORa 2.01, 95% CI_ca_ 1.35-2.99).

Focus groups of men and women in the two districts confirmed the findings from the household survey. Most groups in the SM district considered services had deteriorated, while views in the NGO district groups were more mixed.

“Health facilities are deteriorating on a daily basis.” (Male group, the SM district)

“The attitude of doctors has considerably improved now. They examine us better as well.” (Female group, the NGO district)

About half the household respondents in both districts said they would be willing to pay, or to pay more, for improved health services: 52% (836/1595) in the SM district, and 46% (722/1573) in the NGO district. Multivariate analysis in both districts indicated that less poor households were more likely to be willing to pay for improved services; in the SM district, households with enough food in the last week were four times more likely to be willing to pay (ORa 4.55, 95% CI_ca_ 2.75-7.53) and in the NGO district households with enough food were also more likely to be willing to pay (ORa 1.62, 95% CI_ca_ 1.02-2.59).

Table 2 shows household perceptions about the change in level of corruption in health services over the last 12 months in the two districts, among those respondents who gave an answer to this question. Rather more respondents in the SM district than in the NGO district thought corruption was increasing. When we asked respondents about the main forms of corruption they were aware of in health services, the majority (71% in the SM district, 64% in the NGO district) were not able or not willing to specify a type of corruption they were aware of. A minority (4% in the SM district, 22% in the NGO district) mentioned unofficial payments or bribes.

**Table 2:** Households’ opinions about changing levels of corruption in health services over the last 12 months in the two districts

Perceived change in level of corruption	Number (weighted %) of household respondents	
	SM district (n=1497)	NGO district (n=1558)
Increasing	266 (18)	120 (8)
Staying the same	480 (32)	600 (39)
Decreasing	383 (26)	422 (27)
Don't know	368 (25)	416 (26)

In SM district government health services were provided mainly by contracting the Provincial Health Office under the Strengthening Mechanism; in NGO district the services were provided mainly by a contracted NGO.

When asked if anyone in the household had ever been asked or expected to make an unofficial payment for health care, some respondents declined to answer: 21% (349/1639) in the SM district and 11% (185/1644) in the NGO district. Of those who responded, 85% (1097/1290) in the SM district and 74% (1078/1459) in the NGO district specifically denied anyone in the household had ever been asked to make an unofficial payment. Some 15% (193/1290) in the SM district and 26% (381/1459) in the NGO district admitted that someone in the household had been asked to make a payment or were unsure if someone had been asked.

Many of those households who reported ever being requested to make an unofficial payment said the last time was within the last month, and most said it was within the last six months (SM district 78% (24/31) and NGO district 76% (189/249)). In the SM district, the mean unofficial payment requested on the last occasion was 930.73 (SD 3065.0, n24) Afghani (US$ 19), with a minimum of 10 (US$ 0.2) and a maximum of 15000 (US$ 299). In the NGO district, the mean amount requested was 253.67 (SD 252.7, n336) Afghani (US$ 5), with a minimum of 5 (US$ 0.1) and a maximum of 2000 (US$ 40).

Participants of the focus groups of men and women suggested that the level of corruption in health services, particularly in government health services, was probably much higher than the findings of the household survey indicated. They said that, in fact, corruption was an everyday experience in the society, and this was reflected in corruption in health services, but they felt powerless to do anything about the corruption. They said people were loath to talk about corruption in general and their experiences of it in particular, for fear of the consequences.

“We are afraid the doctor would turn against us if we were to say anything about this.” (Male group, the SM district)

“The entire country is corrupt. Doctors are part of it.” (Male group, the SM district)

“Some people might think that you people are from the intelligence services. They are afraid of telling you the truth.” (Female group, the NGO district)

“The entire government is corrupt. Nothing will happen even if we do complain about it.” (Male group, the NGO district)

### Household use of health services

Nearly two thirds (63%; 1003/1587) of household respondents in the SM district, adjacent to Kabul, reported household members usually went to a government health facility to seek help for a health problem, while 36% (569/1587) said they went to private health facilities. In the NGO district, further from Kabul, nearly all (93%; 1524/1630) household respondents said household members usually went to a government health facility, and just 5% (87/1630) said they went to private health facilities. In the SM district, in urban sites, taking other factors into account in multivariate analysis, households with enough food in the last week (less poor households) were *less* likely to choose a government facility (ORa 0.30, 95%CI_ca_ 0.27-0.33), as were households where the head had some education (ORa 0.49, 95% CI_ca_ 0.43-0.56). In the SM district rural sites, households were *less* likely to choose a government facility if the head had some education (ORa 0.50, 95% CI_ca_ 0.26-0.96), and they were *more* likely to choose a government health facility if there was one within 5 Km of the community (ORa 1.38, 95% CI_ca_ 1.05-1.82).

Focus groups in the SM district pointed to the inadequacies of government facilities as the reason for choosing private facilities, and they complained about the attitude of doctors in government facilities. In the NGO district, focus groups indicated people went to government rather than private facilities because they could not afford the costs of private care, not because the service at government facilities was good.

“Government health facilities don't offer medicine, competent doctors, gynaecologists, and ambulance services. And the doctors are not punctual. That's why we go to a private clinic.” (Female group, the SM district)

“If I had money I would not have gone to the government clinic. Government clinics are for the poor only.” (Male group, the NGO district)

### Satisfaction of health service users

Nearly all the households who responded to the question in the SM district (97%; 1515/1560) and the NGO district (96%; 1414/1476) said at least one household member had used health services within the last six months. As expected from the findings (above) about usual type of facility used, in the SM district, just over half (58%; 905/1550) of households used a government health facility the last time a household member sought help for a health problem, 40% (625/1550) used a private facility, and 1% (10/1550) used traditional healers. In the NGO district, by contrast, nearly all (92%; 1472/1605) of households used a government health facility the *last time* a household member sought help for a health problem, just 7% (110/1605) used a private facility, and 1% (22/1605) used traditional healers. Among health service users in the last six months, the majority were female: 59% (833/1459) in the SM district, and 74% (1030/1386) in the NGO district. In 43% (638/1497) of reported visits in the SM district and 36% (501/1380) of visits in the NGO district the person for whom help was sought was aged less than five years.

Table 3 shows three aspects of service ratings by users: rating of the reception from the service provider; rating of the quality of care compared with expectations; and overall satisfaction with the service. Ratings by government service users were similar between the two districts. Private service users generally gave higher ratings than government service users, although the small number of private service users in the NGO district gave lower ratings for some aspects. Table 4 shows the variables related to three elements of service rating, from the final multivariate models. “District” was not significantly associated with any of the aspects of service rating. A user of a government facility was less likely to rate their reception by the health worker as good or very good than a user of a private facility, while service users in urban sites and those from less poor households were more likely to rate their reception positively. A user of a government health facility was less likely to report the care was better than expected, and less likely to be satisfied with the overall care. Among users of government health facilities, service users were less likely to be satisfied overall if they were from a household with an educated head. They were more likely to be satisfied overall if their household had enough food in the last week, if they did not have to wait more than 30 minutes to be seen, and if they were in an urban community.

**Table 3:** Views of health service users in the two districts about the quality of the service they received from government and private facilities

Views of service users	Proportion (weighted %) of service users	
	SM district	NGO district
*Government service users*		
Reception from health worker good or very good	671/836 (80)	968/1258 (76)
Quality of care better than expected	312/837 (37)	604/1257 (48)
Satisfied with overall care	563/841 (67)	845/1258 (67)
*Private service users*		
Reception from health worker good or very good	557/581 (96)	92/99 (93)
Quality of care better than expected	396/580 (68)	36/100 (35)
Satisfied with overall care	487/583 (84)	46/99 (47)

In SM district government health services were provided mainly by contracting the Provincial Health Office under the Strengthening Mechanism; in NGO district the services were provided mainly by a contracted NGO.

**Table 4:** Variables related to ratings by service users in the two districts combined, from multivariate models

Rating elements and associated factors	Adjusted Odds Ratio (ORa)	Cluster adjusted 95% confidence interval (CIca)
(a) Among all service users		
*Rated reception by health worker as good or very good*		
User of government health facility	0.18	0.10-0.35
Urban sites	4.10	1.50-11.19
Household had enough food in last week	1.61	1.18-2.20
*Care better than expected*		
User of government health facility	0.45	0.33-0.61
*Satisfied with overall care*		
User of government health facility	0.56	0.40-0.80
(b) Among users of government health facilities		
*Satisfied with overall care*		
Household head has some education	0.67	0.53-0.86
Household had enough food in the last week	1.61	1.32-1.98
Waited less than 30 minutes to be seen	2.32	1.57-3.44
Urban sites	2.24	1.01-4.93

### Costs of using health services

Government health service users did not get a free service in either of the two districts. Apart from travel costs to reach the facility, paid by 49% of service users in the SM district and 77% of service users in the NGO district, most service users made payments both within the facilities, and outside the facilities to pay for medicines and investigations. Table 5 summarises the payments reported by government health service users in the two districts. A minority paid a small amount for a “ticket” or registration fee, and about the same small proportion paid a larger amount for their treatment or investigations in the facility. Most of the service users in both districts paid for medicines or further investigations outside the government facilities, and this was generally their biggest expense. The reported median amounts paid within the government facilities were similar to the reported median amounts paid in private facilities (100 Afghanis, US$ 2), and the median amounts paid outside the government facilities approached those reported by users of private facilities (300 – 350 Afghanis, US$ 6-7).

**Table 5:** Payments made by government health service users in the two districts

Item	Proportion (weighted %) paying and median amount in Afghani (US$)*	
	SM district	NGO district
“Ticket” or registration fee	82/838 (10) 5.00 (0.1)	386/1262 (31) 5.00 (0.1)
Treatment and investigations in facility	113/838 (14) 100.00 (2)	211/1262 (17) 100.00 (2)
Medicines etc outside the facility	695/832 (83) 200.00 (4)	895/1249 (72) 250.00 (5)

*The median amount is among those who paid anything.

In SM district government health services were provided mainly by contracting the Provincial Health Office under the Strengthening Mechanism; in NGO district the services were provided mainly by a contracted NGO.

Focus groups of men and women in the two districts confirmed that “everyone is aware” of the policy of free treatment in government facilities but they were equally clear that this policy was not followed in practice. They described the sorts of things they had to pay for in government health facilities, ranging from a small payment for the ticket, to payments for medicines, investigations and procedures. They were aware that some of these payments were unofficial, essentially bribes. They identified the range of people they might have to pay, from the doorman to the doctor. The groups showed a mixture of resignation, frustration and anger about the payments they had to make. They considered that if they did not pay, they would not receive the treatment.

“They charge 50 Afghanis [US$ 1] for an eye check up.” (Female group, the NGO district)

“We give bribes to the birth attendant in hospitals.” (Female group, the SM district)

“We pay only doctors and caretakers.” (Male group, the SM district)

“We pay all the personnel in the facility.” (Male group, the NGO district)

Group participants explained that payments for using government health facilities could pose a serious strain on family resources. Sometimes they had to take loans or sell assets in order to find the money.

“If money is not available we have to sell our livestock or take loans.” (Male group, the SM district)

## Discussion

The social audit, collecting information about health services from the intended beneficiaries in households, provided important insights to complement facilities-based monitoring [10].

### Limitations

The demonstration social audit covered only two districts, purposively selected, and the findings in these two districts cannot be generalised to the whole country. Access to and experience of health services in districts further from Kabul and more affected by the ongoing conflict are likely to be worse than in the two districts in this study.

Satisfaction ratings are an imprecise measure. Overall household ratings are affected by a complex mixture of factors and are susceptible to rapid changes, for example in response to a negative media report about services or a negative experience of a family member or neighbour. Satisfaction ratings based on a specific recent contact with health services are likely to be less affected than household satisfaction ratings by external factors, but still result from a mixture of personal and service factors, and the interplay between them. Prior experience and expectations make a difference.

### Household perceptions and use of services

Most households relied on government health facilities, run under contract by an NGO or under the SM, for treatment of health problems. A notably higher proportion of households in the SM district (over a third) than in the NGO district (less than 10 percent) reported they usually used a private health facility for treatment of a health problem. This probably reflects the greater access in the SM district to the private facilities in Kabul. In both districts, discussions in focus groups suggested that more households would use private facilities if they could afford to do so, because they believed the quality of care in private facilities would be better. In the SM district, where we could analyse this, the better off households (those with enough food and those with a head that had some education) were indeed more likely to use private facilities. In rural parts of the district, access made a difference, and households were more likely to choose a government facility if there was one nearby. The greater use of private health care by those households that can afford it is in line with the findings of other studies in the region. A 2004-5 survey in Afghanistan also found that better off households were more likely to make use of private health care services [11]. In Pakistan and Bangladesh, nationally representative surveys found that many households chose private health service providers over government facilities, and more said they would do so if they could afford it [12-14].

The rating of available health services by households across the two districts included both government and private services available to them. Those households with enough food in the last week were more likely to rate services positively; this perhaps reflects their greater ability to access services, as well as perhaps better treatment in those services they do access. In the focus group discussions two common themes were “poor people have no choice of service” and “poor people are treated badly in government health services”.

In multivariate analyses examining the effects of both individual and service factors, we attempted to tease out the factors associated with ratings by service users. All ratings were more positive among users of private health facilities. Among government service users, overall satisfaction was related to a mixture of personal factors and services factors: a shorter waiting time increased the chances of being satisfied, and users from less poor households were more likely to be satisfied. The higher satisfaction in urban areas might reflect other service factors that were better in urban clinics than in rural clinics. Interestingly, users from households with a head who had some education were *less* likely to be satisfied; perhaps they look for higher standards. A study based on data collected from primary health care facilities in Afghanistan in 2004 found that poorer service users got better quality of service in NGO-contracted facilities than directly managed government facilities [15].

User ratings of services, put together with information from services themselves, can be a useful barometer over time of how services are performing and serving the needs and wishes of the population. Used carefully, such ratings can be part of the way of assessing the quality of service overall and the quality of service from different service providers.

### Costs of using health services

In a poor country such as Afghanistan, costs of health services are a crucial consideration. Often the main costs considered are those borne by the service providers, yet out of pocket costs to service users are important. They limit access to services by the poorest households, and lead to frustration and resentment among service users and non-users. According to official policy in Afghanistan, services in government health facilities (including those run directly by the MOPH, and those run by the SM or by a contracted NGO) were provided free of charge in 2008. However, in practice those who used government health services in our study often had to bear considerable direct monetary costs, in addition to such costs as loss of work time for daily wage earners (Table 5). The costs were similar between the district with government services run under the SM and the district with government services provided by a contracted NGO, and are comparable with those reported from a previous survey in Afghanistan [11].

In the context of a supposedly free service, the reported costs are considerable in Afghanistan, the poorest country in the Asia and Pacific region and where 42% of the population are below the national poverty line [16]. Although focus groups voiced the perception that poor people are forced to use government services, the costs of treatment and investigations in government facilities, among those who paid, were the same as these costs in private facilities. The costs outside the facility (mainly for medicines) among users of government services were not much less than those incurred by users of private services. The costs were not less for facilities run by contracted NGOs than those run by the provincial health office under the SM. The focus group discussions confirmed that health care costs can place a serious strain on already precarious family finances; people have to take loans or sell livestock to find the money. These findings suggest that a review of *actual* charges in government health facilities is needed, especially as most of these charges are apparently not officially sanctioned.

### Corruption in health services

Although the focus group discussions made it clear that people understood the payments they made in government health facilities were unofficial, household respondents were loath to talk about corruption in health services, declining to answer the questions about this, or responding that they “knew nothing about it”. Especially in the SM district, a sizeable minority of respondents could not or would not answer questions about the types of corruption they knew about, or their own family's experience of being asked for unofficial payments or bribes. The fear associated with talking about corruption that emerged from the focus groups is a cause for concern. This climate of fear and suspicion allows corruption to flourish and can thwart attempts to tackle the problem. Afghanis see corruption in the health services as part of the overall high level of corruption in their country, about which there is considerable international concern [17]. Afghanistan was rated the third most corrupt country in the world in 2010 [18]. On a positive note, tackling corruption in health services is probably easier and less dangerous than tackling corruption in, for example, the police or justice services, and it could provide a starting point for a more general campaign against corruption.

## Conclusions

The social audit process was feasible, at least in the two districts included in this study, and produced some actionable findings. The majority of households used contracted government health services but preferred private services. Although people made unofficial payments in government facilities, run by either the provincial health office or an NGO, households were loath to discuss corruption, fearing the consequences. Tackling corruption in health services is an important part of anti-corruption measures in Afghanistan.

## List of abbreviations used

BHC: Basic Health Centre; BPHS: Basic Package of Health Services; CHC: Comprehensive Health Centre; CIca: Cluster-adjusted Confidence Interval; MOPH: Ministry of Public Health; NGO: Non Governmental Organisation; ORa: Adjusted Odds Ratio; PHO: Provincial Health Office; SM: Strengthening Mechanism

## Competing interests

The authors declare they have no competing interests.

## Authors' contributions

AC supported the study design and data analysis and drafted the manuscript. AK and NMA led the data collection, supported the analysis of focus group data, and contributed to the manuscript. KO supported data collection and contributed to the analysis and the manuscript. CH undertook some of the analysis and contributed to the manuscript. NA designed the study, supported the analysis, and assisted with drafting the manuscript.
